# Aboriginal and Torres Strait Islander Attitudes to Organ Donation in Central Australia: A Qualitative Pilot Study

**DOI:** 10.1097/TXD.0000000000001692

**Published:** 2024-08-29

**Authors:** Paul Secombe, Emslie Lankin, Rosalind Beadle, Greg McAnulty, Alex Brown, Michael Bailey, Rebecca Schultz, David Pilcher

**Affiliations:** 1 Intensive Care Unit, Alice Springs Hospital, Alice Springs (Mparntwe), NT, Australia.; 2 School of Medicine, Flinders University, Bedford Park, SA, Australia.; 3 School of Public Health and Preventive Medicine, Monash University, Melbourne, Vic, Australia.; 4 Australian and New Zealand Intensive Care Society Centre for Outcome and Resource Evaluation, Prahran, Vic, Australia.; 5 Centre for Remote Health, Flinders University, Alice Springs (Mparntwe), NT, Australia.; 6 National Centre For Indigenous Genomics, College of Health and Medicine, Australian National University, Canberra, ACT, Australia.; 7 Aboriginal Health Equity, South Australian Health and Medical Research Institute (SAHMRI), SA, Australia.; 8 Public and Primary Health Care, Alice Springs (Mparntwe), NT, Australia.; 9 Department of Intensive Care, The Alfred Hospital, Prahran, Melbourne, Vic, Australia.

## Abstract

**Background.:**

Organ transplantation is a well-established intervention but is reliant on the donation of organs and tissues, mostly from deceased donors. The proportion of Australians proceeding to organ donation (OD) has increased, but the proportion of Indigenous Australians proceeding remains two-thirds that of non-Indigenous Australians. We sought to explore perceived barriers and enablers for the involvement of Indigenous peoples in the OD process.

**Methods.:**

Qualitative methodology centered around focus groups was used to capture the experiences and perspectives of Indigenous people regarding OD. A purposively sampled group of Aboriginal Liaison Officers working within the Alice Springs Hospital Intensive Care Unit (ASH ICU) participated in up to 6 focus groups during 2021 with subsequent thematic analysis of the enablers and barriers to Indigenous participation in the OD process. The ASH ICU is the only ICU servicing Central Australia, and 70% of admissions are Indigenous patients.

**Results.:**

Four primary themes emerged: OD is a new and culturally taboo topic; conversations related to OD are confronting; education is needed (both about OD and cultural education for clinicians); and lack of trust in the healthcare system.

**Conclusions.:**

There are cultural barriers to engaging in the OD process and clinicians need more training on the delivery of culturally safe communication is needed. Despite this, there was a recognition that OD is important. Education about OD needs to be place based, culturally and linguistically appropriate, informed by local knowledge, delivered in community, and occur before a family member is admitted to ICU.

Dying “off-country” is like removing a book from a bookshelf and never putting it back. (HW)

Organ transplantation is a highly effective and well-established intervention that saves lives and improves the quality of life for many Australians. It is cost-effective when compared with alternative treatment options.^1-3^ Transplantation requires the donation of organs and tissues, which, in Australia, is predominately after death. Considerably, more patients wait for a transplant than there are donor organs available.

The 2008 Australian Government national program to implement a world’s best practice approach to increase organ and tissue donation for transplantation culminated in the formation of the DonateLife network and increased the number of organ donors and the number of organs transplanted over the subsequent decade.^1^ The DonateLife network oversees a national program coordinating organ donation (OD) agencies in all states and territories and the placement of specialist nursing and medical personnel in hospitals across the country.^4,5^ It is thought that this network is responsible for increasing the consent rate (the number of families consenting to proceed with OD divided by the number of requests made); however, there are significant differences in the consent rate according to ethnic or cultural group, such that minority groups (including Indigenous Australians) having consent rates much lower than the national average.^6^

The Northern Territory (NT) OD consent rate is 9%, compared with a national average of 54%.^5^ The proportion of Indigenous patients in NT intensive care units (ICUs) is greater than in other jurisdictions, and the Indigenous family consent rate for OD is approximately two-thirds that of non-Indigenous Australians.^6-8^ Although there is speculation about the reasons for this, there has been little published in this area.^9^ Moreover, an Indigenous voice continues to be largely absent in informing the donation community about how this should be approached.^9,10^

Although multiple organs are potentially donated for the Aboriginal and Torres Strait Islander people of Australia (hereafter respectfully Indigenous), kidney transplantation is of particular interest. Like many First Nations peoples internationally, Indigenous Australians bear a disproportionate burden of chronic kidney disease (CKD), ultimately manifesting as dialysis dependent CKD (chronic kidney disease stage 5 dialysis [CKD5D]).^11-16^ Although there is evidence that there are access disparities to kidney transplantation between Indigenous and non-Indigenous patients with CKD5D, the Australian Government-funded National Indigenous Kidney Transplantation Taskforce has undertaken work in improving access to transplantation waitlists, and in ensuring culturally safe care is delivered within transplant units.^17-19^ Although significant work is being undertaken to improve access to transplantation, there remains a deficit of well-matched organs.

We undertook this pilot qualitative study to investigate first the feasibility of undertaking qualitative work on this topic and second to understand better the knowledge of and attitudes to OD among a group of Central Australian Indigenous health workers.

## MATERIALS AND METHODS

This study was conducted in the Alice Springs Hospital (ASH) ICU, a 10-bed ICU in Central Australia. It is the only critical care facility for 1500 km in any direction, serving a population of approximately 50 000 dispersed over 1 million square kilometers, including approximately 50 major remote Indigenous communities of varying sizes and with a diverse range of local languages and affiliations. Central Australia is a loose geographical term used to encompass the southern expanse of the NT, the northern parts of South Australia, parts of WA that are alongside the western border of the NT, and parts of Queensland that border the southeastern border of the NT.^20^ A significant number of residents of the catchment area experience profound socioeconomic disadvantage, with correspondingly poor social determinants of health.^21,22^ Approximately two-thirds of the patients admitted to the ICU identify as Indigenous.

ASH employs Aboriginal Liaison Officers (ALOs) who provide patient advocacy, education, interpretation, and support for Indigenous inpatients and their families. They frequently act as intermediaries between patients and healthcare staff.^23^ Individual members of the ALO team contribute to cultural and linguistic competency, encompassing the diversity of Central Australian Indigenous communities (**Figure S1, SDC,** http://links.lww.com/TXD/A688). ASH ALOs, selected by purposive sampling, were invited to participate in a series of six 1-h focus groups between May and November 2021.^24^ Focus groups were held in a building removed from the hospital campus, allowing separation from the workplace. Included in each focus group was a qualitative researcher (R.B.) and an Aboriginal Health Practitioner (E.L.), who also acted as a cultural broker facilitating navigation of and ensuring cultural safety during the focus groups. The extended time frame allowed for fatigue mitigation, provided the time and space to fill in gaps in knowledge about the topic, increased familiarity and trust between participants and researchers, and allowed for reflexivity between sessions.

Focus group conversations were analyzed to capture some of the experiences and perspectives of Indigenous people in relation to OD in Central Australia. Participants were encouraged to describe and discuss their ideas alongside and among peers and were led by a facilitator (R.B.) and a cultural broker (E.L.). DonateLife medical specialists were invited to the groups to clarify technical matters or knowledge gaps.

Refreshments were provided, and participants received a small gift voucher acknowledging their time contribution. Recognizing that, for Indigenous people, narratives are “fundamentally social and relational” and are often “the product of collaborative lives,” participants were encouraged to invite others to the group as needed (snowball sampling) to increase the authority of the discussion.^25,26^

Conversations were mainly in English but local languages were also used. The members of the group came from diverse language groups (**Table S1, SDC,** http://links.lww.com/TXD/A688), and, for some, English was a second or third language.

The research group, including the cultural broker, developed a number of possible introductory questions using a Delphi-like process to arrive at a single leading question posed by the first focus group: “What has been your experience of OD?” Subsequent focus group sessions commenced with a recap of the previous session, and discussion followed from the recap.

Conversations were recorded and transcribed *verbatim*. The facilitator and cultural broker together undertook thematic analysis using an inductive, latent approach to produce broad themes, which were further divided into subthemes. Thematic analysis is a useful tool to aid interpretation of “patterned meaning” across the range of concepts that appear in a discourse.^27^ Participants reviewed identified proposed themes and verified them at a final respondent validation meeting.

The positionality of researchers is important in qualitative research. Two of the research teams identify as Aboriginal; one is an Aboriginal Health Practitioner who also acted as a cultural broker. Six of the research groups have lived and worked in Central Australia for prolonged periods. Further information on the positionality of each author is included in **Table S2 (SDC,** http://links.lww.com/TXD/A688).

Ethical approval was granted by the Central Australian Human Research Ethics Committee (CA-21-3969). The study is reported following the Consolidated Criteria for Reporting Qualitative Research standards (**Table S3, SDC,** http://links.lww.com/TXD/A688).^28^

## RESULTS

**FIGURE 1.**
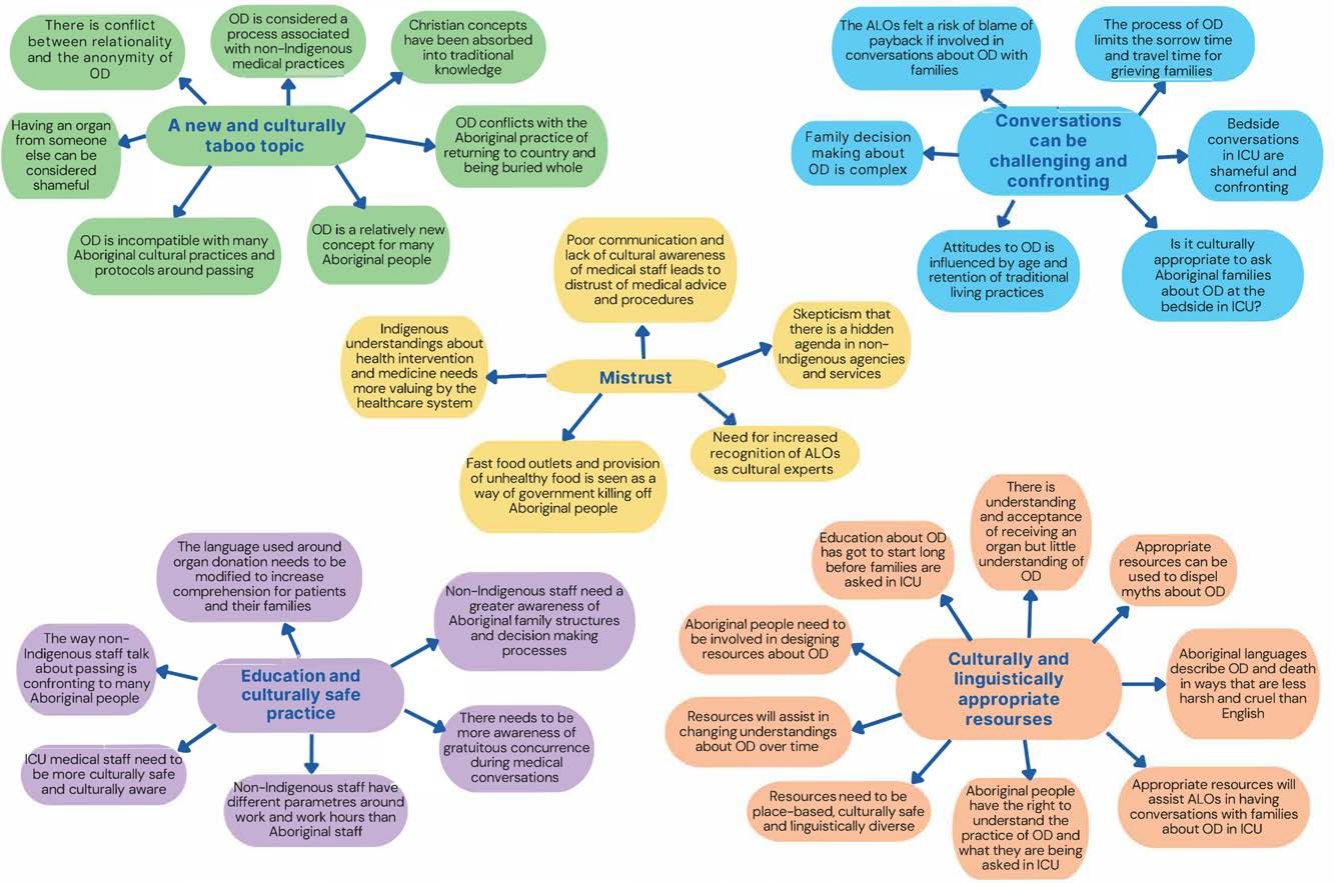
Thematic Analysis Chart. ALO Aboriginal Liaison Officer; ICU, intensive care unit; OD, organ donation.

Nine ASH ALOs variably attended 6 focus group meetings (**Table S1, SDC,** http://links.lww.com/TXD/A688) and 1 respondent validation session. The median age was 46 y (interquartile range, 41–51), and 4 were women. A number of language groups and geographical areas surrounding Alice Springs (Mparntwe) were represented (**Table S1 and Figure S1, SDC,** http://links.lww.com/TXD/A688). ALOs made clear that they represented many viewpoints, including those of family and community, as well as health professionals, acknowledging that their knowledge of the health system was generally more developed. We identified and confirmed many interrelated themes, all of which were weighed similarly by the ALOs (Figure 1). Four overarching themes are presented, and for further details, see **Detailed Thematic Analysis (SDC,** http://links.lww.com/TXD/A688).

### OD is a New and Culturally Taboo Topic

(I)t’s never been spoken about in the past with our mob. (HW, FG 5, 2021)

Due to the high prevalence of CKD5D and the complexity of social connections, all group members had family or friends on regular dialysis or who had received a (renal) transplant. They acknowledged that the concept of OD was still relatively new for Indigenous people in Central Australia.

In the past—when our people were walking around—we never had organ donation … it’s never been spoken about in the past with our mob. For our people it’s a new thing—[the] only organs we know is when we go out hunting that come from animals, it’s a totally new thing. (HW, FG 4, 2021)

The concept of donating organs after death was seen as confronting and culturally taboo. There was, in addition, perceived conflict with the Indigenous practice of returning to the country and being buried whole.

It’s sort of taboo really amongst our people so we need to be careful about what we’re treading on. (HW, FG 4, 2021)

…so he can’t half *ult-antama tinchultumay* (a person can’t be buried half empty). They take everything with them; they don’t want to leave things here. (JA, FG 2, 2021)

This concept was further illustrated by an anecdote about an amputee.

Part of his leg was chopped off and he said, “No, no, no. No you can’t take it. I’ve got to go back for a funeral.” And I’m thinking, “What funeral?” He said, “I’m going to bury my leg first, have a funeral for my leg.” Because when you look at it from the Aboriginal perspective our body is part of the land. Whatever we are going to be giving, [we are] giving away a piece of something which we relate to that land and when we die our body goes back to the land and our spirits roam free on the land. That’s who we are. (HW, FG 1, 2021)

Attitudes to OD varied and appeared to be influenced by age and maintenance of traditional living practices. It was thought that attitudes toward OD may differ across different generations.

They [the elders] told me, “It’s a tricky one, organ donation is not our thing,” but on the other hand, some of them recognised the value in my involvement in finding ways to address the high rates of chronic disease and renal dialysis in our community. (EL, FG 1, 2021)

It was observed on several occasions that traditions change and incorporate external ideas and 2 specific examples were provided. First, in observing how Western religious concepts have been incorporated into Indigenous knowledge.

(Y)ou know every Aboriginal community we preach God, unexpected death happens at home you know and it wasn’t meant to happen. We see that family member pass away early in the morning when we wake up for breakfast and I know that I can say this and you know we all say it oh it was God’s plan you know and we accept it, oh it was God’s choice, she passed away in her sleep peacefully. (EL, FG 4, 2021)

“I know our mob we’re coming back you know from generations from very, very strong Aboriginal communities and law and culture and all that but nowadays, today we’re all about God you know, we pray and we pray.” (EL, FG 4, 2021)

It was further observed that there were mixed messages from different Christian groups, including one denomination, which appears to be actively deterring people from seeking medical intervention, including dialysis, organ transplants, and OD.

Yeah, we [medical staff] want to give you [a new] kidney and dialysis and she said, “No, Jesus told me not to look for dialysis.” They [community members] were being told by the church not to go on dialysis because God told them not to. The Church tells them, “You can’t go and get a kidney or anything like that because you’re not supposed to look for medicine.” Because of the church … she went and passed away and she was only in her 20s and she had a little baby and, you know, it’s because of the church’s influence. (CS, FG 4, 2021)

Second, when observing how local Indigenous attitudes to blood transfusion have changed.

…having somebody else’s blood could make them sick. It scared the whole community … [but] they accept it now [that there is better information about it]. (CS, FG 6, 2021)

### Confronting Conversations

Families might get angry if they get asked like that. (JA, FG 2, 2021)

Unexpected conversations with families about OD may result in feelings of shame, confusion, offensiveness, and confrontation. ALOs described the risk of being caught between clinicians and family.

Families might get angry if they get asked for organs like that, especially when they are mourning. They would say, “Get out, what are you hanging around here for?” (JA, FG 2, 2021)

They’d look at you thinking, “Are you mad, are you crazy?” (LB, FG 3, 2021)

The close relationships between ALOs and families were perceived as having mixed consequences. Although there was the advantage of providing familiarity and cultural brokerage, there was concern it also created the perception that ALOs were aligning themselves with “White man’s” practices. This risk was greater with families to which the ALO was related. These were “really uncomfortable” (CS, FG 3, 2021); a “shame job” (LB, FG 3, 2021); and it could be so offensive to the families that “(t)hey could bone us and make us very sick too” (LB, FG 3, 2021).

End-of-life discussions are seen as confronting, compounding mistrust of the healthcare system and assumptions of systemic racism.

They don’t talk like that to the white fella families, with white families they’ll use some other words, not straight out, “You’re going to die.” (LB, FG 5, 2021)

### Cultural Education (Mainly of the Western Medical System)

Because our people need to know. (HW, FG 1, 2021)

Accepted medical practice in breaking bad news (using unambiguous, noneuphemistic terms such as “death” and “dying” early in the conversation) was seen as confronting and offensive for Indigenous people. When medical staff “use that language when the families are going through a really hard time it makes it harder for them.” (CS, FG 5, 2021)

But it’s the language they use too hey, that upsets the family members you know. (CS, FG 5, 2021)

It was reported that Indigenous families prefer that death be referred to in “roundabout ways” (EL, FG 6, 2021).

We would say something like, “Sorry but the doctors can’t do anything for you.” (CS, FG 5, 2021)

Sorry but the medicines can’t help anymore. We [ALOs] interpret that back to the patients or families [in ways that are comfortable for them]. (CS, FG 6, 2021)

The term “organ donation” sounds very “harsh and cruel” when translated into local Indigenous languages (CS, FG 1, 2021). However, the ALOs acknowledged that participation in the focus groups had been educational as well, and the new knowledge acquired during the focus groups informed further discussion and was thought would assist in supporting conversations with Indigenous families in the ICU.

It’s good cause we’re learning ourselves you know and if we can know more about organ donation we can help our people, like talk to them you know. At least we know more. [The] only transplant they know is kidney. (CS, FG 4, 2021)

Furthermore, in identifying that education was a necessary prerequisite to any conversation with family within the ICU, the ALOs identified an absence of locally produced, place based, culturally and linguistically appropriate resources. This in turn led to discussion around the type of resources, which may be effective with local communities, and an engagement by the ALOs in creating these resources.

The conversation starts within the community itself way before coming to ICU. You’ve got to start educating…. At the moment there are no resources out there to [support us to] talk about it. (HW, FG 4, 2021)

The interwoven strands of work, family, and community commitments and the delicate and sensitive position that the ALOs hold in a small community necessitated careful navigation between 2 worlds. Clinicians must be aware of the conflicting allegiances when ALOs are involved in difficult conversations with families.

After work we’ve still got sorry business. After being with people in ICU, they still come to us with questions like, “Where can I go?” or “What can I do for my family member?” They come [to my] home knocking on my door. Our work keeps going after work. (CS, FG 6, 2021)

The mentality of Aboriginal people is a circle. A White man’s perfect world is a square, our circle doesn’t fit into it, you know, it’s just square—they come to work 6 am, knock off 4:21 pm. They use square phones, square laptops, everything is square and when they knock off they go back to the square inside the yard, inside the house. We, we’re still in a circle, you know, when I knock off we go and sit down with family, we mix up with family, we still meet the patients out on the street, we deal with it that way every day. (HW, FG 6, 2021)

This was taken to allude to the perceived difficulties that the ALOs, in particular, face when being engaged in difficult conversations with families who have a critically ill family member in the ICU. While clinicians are (perceived) as having a separate work life and personal life, for the ALOs, work is an extension of their personal life, and their personal life is an extension of their work life. For many of them, conversations (and interpretation) are brokered between clinicians and people who are direct or indirect family members. People that they have known for their entire lives and people that they are likely to have ongoing familial relationships with.

Whether it is culturally appropriate to raise the question about OD with families was questioned.

I think it’s not on cause people get upset, especially in a sorrow (grieving) mode. They don’t want to talk about people’s body getting dismembered … so I think that ICU discussion with the doctors between the family and the patients, that needs to come to a halt at this stage. We need to educate them about organ donation prior [to arriving in ICU]. [Currently] you’re catching people off guard when they’re in sorrow mode and sometimes they’re upset and they don’t want to make a decision. That’s what I’ve come across. (HW, FG 6, 2021)

For a family to be exposed to such information at the bedside during an “end-of-life” discussion is confronting.

(y)ou’ve got to start educating, if you’re going to save four or five lives out of one tissue then that conversation needs to be started. (HW, FG 4, 2021)

### Mistrust

(C)ause you know like we still get people coming in oh they’re going to poison me here or you know things like that. (CS, FG 6, 2021)

On a number of occasions, the theme of mistrust of institutions emerged and was often intertwined with the other themes identified. In particular, there is mistrust around the motives of government, churches, and the healthcare system. This appeared to spill over into concerns about the motives of government programs and policies, with the underlying assumption that there was always a hidden agenda that would further disadvantage Indigenous people.

That’s where this government thinking is wrong. I think they’ve been trying to kill us off for 223 years you know. They’ve found a way of killing our mob. (HW, FG 4, 2021)

Churches have been helping the government to steal the land so what’s the point you know? That’s how I see it. They came along and our people put their head down and prayed and turned around and looked and thought, “Hey where’s my land, it’s gone,” you know? And they were told to work 14 hour shifts to build a yard, build a fence line and next minute they were kicked over the other side of the fence line and told, “You live on this side.” (HW, FG 4, 2021)

(T)hat’s why they don’t have that trust with the community or community doesn’t trust them because of the issues in the past or you know. (CS, FG 6, 2021)

It was thought that this mistrust could be mitigated through education, especially if it was culturally and linguistically appropriate and had local input into its design.

Not just when you’re [patients] sick. You need to educate people before. Educate from the community, we’ve got to educate them. It will happen you know, you never know. Just like that it will happen or something. (BS, FG 1, 2021)

## DISCUSSION

In this pilot qualitative study, the first in the published literature to actively explore Australian Indigenous voices in OD, we have demonstrated that it is feasible and possible to explore a difficult topic through small focus groups. We found a desire for more knowledge about OD, particularly if presented in a culturally and linguistically appropriate manner. Furthermore, although donation is seen as difficult to talk about (and culturally taboo), it is perceived as important. We also found a deep mistrust of Western institutions and the motives of these institutions. This is compounded by the approach to death and dying practiced by many clinicians, which was considered to be culturally inappropriate.

Over the course of the focus groups, there was a tacit acknowledgment that OD was an important topic. What began as an emphatic “no” to the question of whether donation should be raised became more nuanced over the focus groups, perhaps with better information and understanding. Although it had not been the initial intent to have the same group attend sequential focus groups, this occurred organically and was informed by the decision of the ALOs not to take up the invitation to invite other community members (snowballing). It provided the additional advantage of establishing trust and comfort between the individual group members and between group members and the researchers as a difficult topic was explored. This highlights the importance of ongoing engagement and may also be key to improving community acceptance of, or at least the concept of, OD.

The health and cultural importance of the symbiosis of country (land) and people underscored many of the themes. Interconnectedness of people to the country, particularly the importance of returning to the country (especially during the burial process), was woven throughout the focus groups. OD may represent a risk to the normative cultural practice of returning to the country “whole,” which requires further examination.

Cultural and religious elements and societal values and beliefs influence attitudes to OD.^29,30^ Negative beliefs reduce potential organ donor registration, which may influence family consent for donation to proceed.^31^ Our analysis revealed that trust in the healthcare system is low, an oft-reported phenomenon among minority groups internationally arising from previous experience(s) of discrimination.^32-35^ The ALOs suggested that this could be ameliorated through place based, culturally and linguistically appropriate resources codesigned by the community—an activity that they have subsequently pursued. The inclusion of an Indigenous voice in this process is crucial, as was highlighted by the subsequent response to exclusion of First Nation representation in the decision by a Canadian jurisdiction to alter OD consenting methods.^35^ Furthermore, the observation that both religious concepts and ideas around blood transfusion have been incorporated into Central Australian Indigenous culture potentially offers insights into how OD could come to be accepted into local Indigenous ideas of healthcare.

Decisions to register as a potential organ donor and family decisions to proceed to OD are distinct. Family consent to OD exceeds 80% in the general population when the potential donor is registered on the Australian Organ Donor Register.^5^ As a result, much research focuses on factors influencing registration rather than those for consent rates in ICU.^36^ Existing data support the role of education outside the ICU.^31^ Our analysis suggests that this is also true for Central Australian Indigenous people.

Studies of Indigenous attitudes to OD are notably underrepresented in the Australian literature on donation among minority groups. Notably, there is literature examining attitudes among Arabic speakers, as well as Serbian, Macedonian, and Greek Orthodox communities, yet the voices of the First Nations people of Australia have been largely unrepresented.^37,38^ The deep level of mistrust that emerged in several themes is likely to reflect generations of systemic racism and failures of the healthcare system to adequately listen to and respond to the voice of Indigenous Australians.^39,40^

A narrative review by Cairnes et al and a gray literature report exploring Indigenous attitude to OD in Western Australia (WA) are exceptions.^41,42^ There is a remarkable correlation between the themes identified in our data and the themes identified in the WA report. In particular, the desire for community-based education and discussion to prepare families, such that the bedside of the dead or dying family member is not the setting for the introduction of the concept of OD.^9,41^ Furthermore, clinicians need a better understanding of culturally safe communication when embarking on these discussions.

International literature on First Nations people and OD reflects several of our themes. Commonalities that were found to exist included an emphasis on avoiding dismemberment or interference with the body and the importance of being “buried whole.”^43-46^ There was little in the international literature that focused on a return to country, which was clearly important among Central Australian Indigenous people. The theme of healthcare mistrust has been extensively reported among minority groups internationally and, while not unique for Central Australian Indigenous people, appears to underpin some of the resistance by families when approached with an OD request.^32-35^ Similarly, the need for OD to be discussed at the community level echoes similar calls from Australian and International groups.^35,42,44,47-49^

In particular, the international literature reports the tension between maintaining and preserving traditional cultural beliefs and the perceived conflict between these beliefs and the processes required for OD to proceed.^48^ The paradox between acknowledging the advantages that flow from OD (through increased transplantation rates) among First Nations groups, who carry a high burden of dialysis-dependent renal disease, and the apparent conflict with traditional culture means that pathways to explore OD exist.^49^ Knowledge about OD and the OD process was limited in our group and this too is reflected in the international literature.^43,49^

Many First Nations and other vulnerable populations are overburdened with dialysis-dependent renal disease (CKD5D), and many families either know of someone with a transplant or someone who is in need of one.^48^ Interest in receiving a transplant is high among First Nation CKD5D patients, and the National Indigenous Kidney Transplantation Taskforce has introduced a number of initiatives to reverse the inequity in transplant access.^19,39^ However, there remains a substantial deficit in organs for those on transplant waiting lists, and OD rates are consistently low.^5^ Many First Nations populations, including Australian Indigenous peoples, have a unique HLA pattern and an organ from a non-Indigenous donor is likely to be less well matched.^5,7,50-52^ Indigenous people in Central Australia have among the highest rates of CKD5D in the country, if not the world, and, as a group, have the potential to benefit enormously from improvement in donation and transplantation rates.^17,53-55^

### Strengths

We involved community representatives in the form of ALOs employed at ASH. Although the focus groups ultimately explored the views of a relatively small group of Central Australian Indigenous people, the ALOs have family and cultural ties to the local area and are highly respected within the Indigenous community. Our use of a cultural broker working alongside the facilitator naturally led to capacity building among Indigenous health staff.

The qualitative methodology allowed data collection in a cyclical manner such that new insights and experiences led to adaptation and expansion of the original plan, and allowed us to move from a quantitative analysis of what is not working toward an understanding of what may work and why.^56^ The regular and consistent participation of the ALOs demonstrated the appropriateness of this methodology and was further confirmed by the disappointment expressed when the sessions ended.

We’ve been on other focus groups a lot, could take or leave them, but from this, you know, we’re all learning. (CS, FG 5, 2021)

The 2-way nature of our inquiry meant that the research team clinicians learned as much about cultural safety as focus group participants learned about OD. This has resulted in initiatives to improve cultural safety generally and to produce place based, culturally and linguistically appropriate educational materials for Central Australia.

### Limitations

Available resources restricted the collection of thematic data to 6 focus groups (plus a validation session), and it was unclear whether we achieved thematic saturation. Although our themes were derived from the views of Central Australian Indigenous people, as consistencies with work from WA suggest, there may be some transferability across different regions of Australia.^41^ In addition, hospital-based ALOs (as they observed themselves) are likely to be more health literate, and they may not truly represent the degree of understanding of the majority of community members nor be reflective of the views of Indigenous Australians in different levels of urbanization.

Further limitations include the observation that focus groups were conducted in English, which is a second, third, and sometimes fourth language for the participants. In addition, there is also the possibility that some of the themes that emerged were skewed by individual experiences—for example, it would not be standard clinical practice to claim “straight out ‘You’re going to die’” (LB, FG 5, 2021). Although this may reflect a lived experience, even by the standards of using clear noneuphemistic language, it would not be an approach condoned by current clinical practice.

## CONCLUSIONS

Culturally safe and appropriate information is vital to improve knowledge about OD among Central Australian Indigenous people. The effect of the paucity of culturally contextualeducational resources and inappropriate language used by clinicians describing death and dying may exacerbate the sense of mistrust of the Western healthcare system.

Our data highlight the difficulty of engaging in the OD process both for clinicians and Central Australian Indigenous families despite an acknowledgment that it is important. There was a consensus communities need appropriate accessible information well before a loved one has been admitted to the ICU with the potential to be an organ donor. A bedside setting for the delivery of OD information is destructive of trust and we must develop and deliver place based, locally codesigned, culturally and linguistically appropriate educational materials and restore trust in the healthcare system for Indigenous patients.

## Supplementary Material



## References

[1] DonateLife. Progressing Australian organ and tissue donation and transplantation to 2020: the 2016–2020 strategy. Available at https://www.donatelife.gov.au/sites/default/files/2021-05/2016-2020_strategic_plan.pdf. Accessed July 28, 2024.

[2] Altinors N Haberal M. The economics of organ transplantation. Exp Clin Transplant. 2018;16(Suppl 1):108–111. 29528004 10.6002/ect.TOND-TDTD2017.P1

[3] Axelrod DA Schnitzler MA Xiao H. An economic assessment of contemporary kidney transplant practice. Am J Transplant. 2018;18:1168–1176. 29451350 10.1111/ajt.14702

[4] Rakhra SS Opdam HI Gladkis L. Untapped potential in Australian hospitals for organ donation after circulatory death. Med J Aust. 2017;207:294–301. 28954604 10.5694/mja16.01405

[5] Australian Government. Australian Organ and Tissue Donation and Transplantation Authority. Australian Donation and Transplantation Activity Report 2022. Available at https://www.donatelife.gov.au/sites/default/files/2023-02/OTA%202022%20Donation%20and%20Transplantation%20Activity%20Report.pdf. Accessed March 18, 2023.

[6] DonateLife. 2016 Australian donation and transplantation activity report: part 1 organ donation and transplantation. Available at https://www.donatelife.gov.au/sites/default/files/2021-05/australian_donation_and_transplantation_activity_report_2016.pdf. Accessed July 28, 2024.

[7] Secombe P Brown A McAnulty G. Aboriginal and Torres Strait Islander patients requiring critical care: characteristics, resource use, and outcomes. Crit Care Resusc. 2019;21:200–211. 31462207

[8] Waller KMJ Hedley JA Rosales BM. Effect of language and country of birth on the consent process and medical suitability of potential organ donors; a linked-data cohort study 2010-2015. J Crit Care. 2020;57:23–29. 32014644 10.1016/j.jcrc.2020.01.025

[9] Cairnes S Wood L Thomas J. The Views of First Nations People, Including First Nations Australians, on Organ Donation: A Multi-National Perspective. vol 30. Cambridge Publishing; 2021:7–12.

[10] Wakefield CE Reid J Homewood J. Religious and ethnic influences on willingness to donate organs and donor behavior: an Australian perspective. Prog Transplant. 2011;21:161–168. 21736247 10.1177/152692481102100213

[11] Gao S Manns BJ Culleton BF; Alberta Kidney Disease Network. Prevalence of chronic kidney disease and survival among Aboriginal people. J Am Soc Nephrol. 2007;18:2953–2959. 17942955 10.1681/ASN.2007030360

[12] Jha V Garcia-Garcia G Iseki K. Chronic kidney disease: global dimension and perspectives. Lancet. 2013;382:260–272. 23727169 10.1016/S0140-6736(13)60687-X

[13] Dyck RF Jiang Y Osgood ND. The long-term risks of end stage renal disease and mortality among First Nations and non-First Nations people with youth-onset diabetes. Can J Diabetes. 2014;38:237–243. 24986804 10.1016/j.jcjd.2014.03.005

[14] Jiang Y Osgood N Lim HJ. Differential mortality and the excess burden of end-stage renal disease among First Nations people with diabetes mellitus: a competing-risks analysis. CMAJ. 2014;186:103–109. 24295857 10.1503/cmaj.130721 PMC3903736

[15] Maple-Brown LJ Hughes JT Ritte R. Progression of kidney disease in Indigenous Australians: the eGFR follow-up study. Clin J Am Soc Nephrol. 2016;11:993–1004. 27076636 10.2215/CJN.09770915 PMC4891751

[16] Huria T Pitama SG Beckert L. Reported sources of health inequities in indigenous peoples with chronic kidney disease: a systematic review of quantitative studies. BMC Public Health. 2021;21:1447. 34301234 10.1186/s12889-021-11180-2 PMC8299576

[17] Khanal N Lawton PD Cass A. Disparity of access to kidney transplantation by indigenous and non-indigenous Australians. Med J Aust. 2018;209:261–266. 30208818 10.5694/mja18.00304

[18] Cundale K McDonald SP Irish A. Improving equity in access to kidney transplantation: implementing targeted models of care focused on improving timely access to waitlisting. Med J Aust. 2023;219:S7–S10. 37839027 10.5694/mja2.52099

[19] McDonald SP Cundale K Owen KJ. Equitable access to kidney transplants for Aboriginal and Torres Strait Islander people in Australia. Nat Rev Nephrol. 2023;19:751–752. 37857764 10.1038/s41581-023-00780-3

[20] Robson B McAnulty G Secombe P. Critical care resource use associated with tourism in Central Australia. Aust J Rural Health. 2021;29:408–416. 34085730 10.1111/ajr.12737

[21] Tong SY van Hal SJ Einsiedel L; Australian New Zealand Cooperative on Outcomes in Staphylococcal Sepsis. Impact of ethnicity and socio-economic status on *Staphylococcus aureus* bacteremia incidence and mortality: a heavy burden in Indigenous Australians. BMC Infect Dis. 2012;12:249. 23043704 10.1186/1471-2334-12-249 PMC3548680

[22] Zhao Y You J Wright J. Health inequity in the Northern Territory, Australia. Int J Equity Health. 2013;12:79. 24034417 10.1186/1475-9276-12-79 PMC3847185

[23] Grant R Draper N. The importance of Indigenous health liaison officers and family meetings to improve cardiovascular outcomes in Indigenous Australians. Aust N Z J Public Health. 2018;42:499–500. 30151850 10.1111/1753-6405.12824

[24] Patton MQ. Two decades of developments in qualitative inquiry: a personal, experiential perspective. Qual Soc Work. 2002;1:261–283.

[25] Goodman LA. Snowball sampling. Ann Math Stat. 1961;32:148–170.

[26] Moreton-Robertson A. Talkin’ Up to the White Woman: Aboriginal Women and Feminism. University of Queensland Press; 2000.

[27] Braun VaC V. Chapter 7. Thematic analysis. In: Coyle ELaA, editor. Analysing Qualitative Data in Psychology. 3rd ed. Sage; 2021:128–147.

[28] Tong A Sainsbury P Craig J. Consolidated criteria for reporting qualitative research (COREQ): a 32-item checklist for interviews and focus groups. Int J Qual Health Care. 2007;19:349–357. 17872937 10.1093/intqhc/mzm042

[29] Moloney G Walker I. Talking about transplants: social representations and the dialectical, dilemmatic nature of organ donation and transplantation. Br J Soc Psychol. 2002;41(Pt 2):299–320. 12133230 10.1348/014466602760060264

[30] Moloney G Sutherland M Bowling A. Don’t forget the context when you are talking about organ donation: social representations, shared mood and behaviour. J Community Appl Soc Psychol. 2020;30:645–659.

[31] Moloney G Upcroft L Rienks S. Respect, interaction, and immediacy: addressing the challenges associated with the different religious and cultural approaches to organ donation in Australia. Exp Clin Transplant. 2020;18(Suppl 2):43–53. 32758119 10.6002/ect.rlgnsymp2020.L7

[32] Armstrong K Ravenell KL McMurphy S. Racial/ethnic differences in physician distrust in the United States. Am J Public Health. 2007;97:1283–1289. 17538069 10.2105/AJPH.2005.080762 PMC1913079

[33] Bazargan M Cobb S Assari S. Discrimination and medical mistrust in a racially and ethnically diverse sample of California adults. Ann Fam Med. 2021;19:4–15. 33431385 10.1370/afm.2632 PMC7800756

[34] Razai MS Kankam HKN Majeed A. Mitigating ethnic disparities in covid-19 and beyond. BMJ. 2021;372:m4921. 33446485 10.1136/bmj.m4921

[35] Tait CL. The rights and interests of First Nations, Métis, and Inuit in debates over deemed consent legislation for deceased organ donation in Canada: calls to action. Lancet Reg Health Am. 2023;18:100414. 36844019 10.1016/j.lana.2022.100414 PMC9950653

[36] Irving MJ Jan S Tong A. What factors influence people’s decisions to register for organ donation? The results of a nominal group study. Transpl Int. 2014;27:617–624. 24617320 10.1111/tri.12307

[37] Phillipson L Larsen-Truong K Pitts L. Knowledge of, beliefs about, and perceived barriers to organ and tissue donation in Serbian, Macedonian, and Greek Orthodox communities in Australia. Prog Transplant. 2015;25:91–99. 25758807 10.7182/pit2015550

[38] Ralph AF Alyami A Allen RD. Attitudes and beliefs about deceased organ donation in the Arabic-speaking community in Australia: a focus group study. BMJ Open. 2016;6:e010138. 10.1136/bmjopen-2015-010138 PMC4735320 26787253

[39] Hughes JT Cundale K Owen KJ. The National Indigenous Kidney Transplantation Taskforce: changing systems to achieve equitable access to kidney transplantation. Med J Aust. 2023;219:356–357. 37838976 10.5694/mja2.52107

[40] Hughes JT Owen KJ Kelly J. Cultural bias in kidney care and transplantation: review and recommendations to improve kidney care for Aboriginal and Torres Strait Islander people. Med J Aust. 2023;219:S11–S14. 10.5694/mja2.52110 37839026

[41] Scrine C Murray R. Addressing aboriginal rates of organ and tissue donation in WA: report on the community awareness grant. Kulunga Research Network. Available at https://www.researchgate.net/publication/257465212_Addressing_Aboriginal_rates_of_organ_and_tissue_donation_in_WA_Report_on_the_Community_Awareness_Grant. Accessed November 8, 2021.

[42] Cairnes S Wood L Thomas J. The views of First Nations people, including First Nations Australians, on organ donation: a multi-national perspective. Transplant J Australas. 2021;30:7–12.

[43] Molzahn AE Starzomski R McDonald M. Aboriginal beliefs about organ donation: some Coast Salish viewpoints. Can J Nurs Res. 2004;36:110–128. 15739940

[44] Irving MJ Tong A Jan S. Factors that influence the decision to be an organ donor: a systematic review of the qualitative literature. Nephrol Dial Transplant. 2012;27:2526–2533. 22193049 10.1093/ndt/gfr683

[45] Jernigan M Fahrenwald N Harris R. Knowledge, beliefs, and behaviors regarding organ and tissue donation in selected tribal college communities. J Community Health. 2013;38:734–740. 23504267 10.1007/s10900-013-9672-2 PMC3706512

[46] Chen AN Chen KF Chang PC. Hindering factors and suggestions related to organ donation decisions: perspective of the Taiwan Ali-Shan Tsou aboriginal tribe. Transplant Proc. 2014;46:1041–1043. 24815122 10.1016/j.transproceed.2014.01.008

[47] Stephens D. Exploring pathways to improve indigenous organ donation. Intern Med J. 2007;37:713–716. 17894767 10.1111/j.1445-5994.2007.01500.x

[48] Davison SN Jhangri GS. Knowledge and attitudes of Canadian First Nations people toward organ donation and transplantation: a quantitative and qualitative analysis. Am J Kidney Dis. 2014;64:781–789. 25172531 10.1053/j.ajkd.2014.06.029

[49] Devitt J Anderson K Cunningham J. Difficult conversations: Australian indigenous patients’ views on kidney transplantation. BMC Nephrol. 2017;18:310. 29020932 10.1186/s12882-017-0726-z PMC5637064

[50] McDonald S. Indigenous transplant outcomes in Australia: what the ANZDATA registry tells us. Nephrology (Carlton). 2004;9:S138–S143. 15601406 10.1111/j.1440-1797.2004.00350.x

[51] Dittmer I Wilkinson M. The causes and importance of racial disparities in pediatric transplantation in New Zealand. Pediatr Transplant. 2014;18:653–655. 25250965 10.1111/petr.12350

[52] Shaw R Webb R. Ka mura ka muri: understandings of organ donation and transplantation in Aotearoa New Zealand. Med Humanit. 2021;47:456–465. 33753461 10.1136/medhum-2020-012038

[53] Li L Guthridge S Li SQ. Estimating the total prevalence and incidence of end-stage kidney disease among aboriginal and non-aboriginal populations in the Northern Territory of Australia, using multiple data sources. BMC Nephrol. 2018;19:15. 29334912 10.1186/s12882-017-0791-3 PMC5769509

[54] Secombe P Chiang PY Pawar B; Alice Springs Hospital Renal-ICU Research Group. Resource use and outcomes in patients with dialysis-dependent chronic kidney disease admitted to intensive care. Intern Med J. 2019;49:1252–1261. 30667144 10.1111/imj.14232

[55] Secombe P Moynihan G McAnulty G. Alice Springs Hospital Renal ICUrg. Long term outcomes of dialysis dependent chronic kidney disease patients requiring critical care: an observational matched cohort study. Intern Med J. 2021;51:548–556. 31990145 10.1111/imj.14764

[56] Busetto L Wick W Gumbinger C. How to use and assess qualitative research methods. Neurol Res Pract. 2020;2:14. 33324920 10.1186/s42466-020-00059-z PMC7650082

