# Supplementary material for: Aboriginal and Torres Strait Islander Attitudes to Organ Donation in Central Australia: A Qualitative Pilot Study
Source: Transplant Direct. 2024 Aug 29;10(9):e1692. doi: 10.1097/TXD.0000000000001692 (PMC11365648; doi:10.1097/TXD.0000000000001692)
Supplement: Supplementary file 1 [file txd-10-e1692-s001.pdf]

Supplementary Data

Supplementary Tables **2**

Table S1: Focus Group Attendance and Cultural/Family/Language Group Affiliation 2

Table S2: Positionality statement 3

Table S3: Standards for Reporting Qualitative Research 5

Supplementary Figures **7**

Figure S1: Indigenous groups representing distinct cultural, language, customs and laws of Central Australia, 7

Supplementary Detailed Thematic Analysis **8**

1. A new topic and culturally taboo: It's a really difficult topic to talk about 8
2. Confronting conversations: Families might get angry if they get asked like that 13
3. Education about organ donation: Our people need to know 18
4. Cultural education (of the western medical system): They work in a square, we work in a circle 21
5. Mistrust: That's why community doesn't trust them 25

References **27**

Supplementary Tables

Table S1: Focus Group Attendance and Cultural/Family/Language Group Affiliation

Focus group Number		1	2	3	4	5	6	7*
	**Language group**							
**ALOs**								
**BS**	**Central Arrernte**	✓		✓				✓
**CH**	**Anmatyerr**			✓		✓	✓	✓
**HW**	**Pitjantjatjara, Yankunytjatjara**	✓	✓		✓	✓	✓	✓
**AD**	**Alyawarr**		✓	✓	✓	✓		✓
**GS**	**Western Arrernte**	✓	✓	✓		✓		
**LB**	**Pitjantjatjara**	✓		✓		✓	✓	✓
**CS**	**Warlpiri**	✓		✓	✓	✓	✓	✓
**JA**	**Southern & Western Arrernte, Pitjatjantjara**	✓	✓	✓	✓		✓	
**SS**	**Western Arrernte**							✓
**Faciliators**								
**EL†**	**Western Arrernte**	✓	✓		✓		✓	✓
**RB**	**Nil**	✓	✓	✓	✓	✓	✓	✓
**Clinicians**								
**PS**	**Nil**	✓				✓	✓	✓

ALO - Aboriginal Liaison Officer
* - focus group 7 constituted the respondent validation session / member checking session
† - EL attended in dual role of joint facilitator / cultural broker and as a member of the focus group as a Western Arrernte woman.

Table S2: Positionality statement

Positionality describes an individualʹs worldview and the position that they adopt about research and its context To aid readers understanding of the positionally of each of the authors, we include this positionally statement.

The first author is a middle aged white male clinician researcher who has been a consultant intensivist for approximately a decade. This time has been spent exclusively working and living in Central Australia. When undertaking clinical activities he often works with the ASH ALO’s. This work has been undertaken as part of a PhD by this author exploring Indigenous critical illness.

The second author, is a middle aged female Western Arranda Aboriginal Health Practitioner with a Diploma of ATSI Primary Health Care Practice. She has worked in remote communities and within the acute care setting. She joined the research team with a preexisting relationship with the participants through both community connections, and her work at the ASH. Having worked in health in the region for 16 years, she has a sound understanding of the health circumstances and health literacy of Aboriginal people in Central Australia. She has worked alongside the ALO’s at times as part of her clinical role.

The third author is a middle aged non-Indigenous woman who has had extensive experience undertaking community-informed qualitative research in a remote and urban Central Australia. She completed her PhD in 2018, the methodology for which was guided by a group of Ngaanyatjarra women from the Central Western desert. She had no previous relationship with the ALOs.

The fourth author is a middle aged white male clinician researcher who has been a consultant intensivist in excess of three decades. He has lived and worked in Central Australia for nearly a decade, also working closely with the ASH ALO’s clinically.

The fifth author is an Aboriginal man of the Yuin Nation who has family connections to Nowra, Wreck Bay and Wallaga Lake on the far south coast of NSW. He is an internationally leading Aboriginal clinician/researcher who has worked his entire career in Aboriginal health in the provision of public health services, infectious diseases and chronic disease care, health care policy and research. He has no previous relationship with the ALOs.

The sixth author is a white male biostatistician who is a secondary supervisor for the first author’s PhD programme. He has no previous relationship with the ALOs.

The seventh author is a middle age white female nurse who has lived and worked in Central Australia for approximately 5 years. She has no previous relationship with the ALOs.

The eighth and final author is a middle aged white male intensivist with active links to Donate Life. He is the primary supervisor of the first author's PhD. He has no previous relationship with the ALOs.

Table S3: Standards for Reporting Qualitative Research

From Tong et al.[1]

		Page no(s).*
**Domain 1: Research team and reflexivity**		
**Personal characteristics**		
1	**Interviewer/Facilitator:** Which author/s conducted the interview or focus group?	p. 6
2	**Credentials:** What were the researcher's credentials? E.g. PhD, MD	Supplementary p. 2
3	**Occupation:** What was their occupation at the time of the study?	Supplementary p. 2
4	**Gender:** Was the researcher male or female	Supplementary p. 2
5	**Experience & training:** What experience or training did the researcher have?	Supplementary p. 2
**Relationship with participants**		
6	**Relationship established:** Was a relationship established prior to study commencement?	Supplementary p. 2
7	**Participant knowledge of the interviewer:** What did the participants know about the researcher? e.g. personal goals, reasons for doing the research	Supplementary p. 2
8	**Interviewer characteristics** What characteristics were reported about the interviewer/facilitator? e.g. Bias, assumptions, reasons and interests in the research topic	Supplementary p. 2
**Domain 2: Study design**		
**Theoretical framework**		
9	**Methodological orientation and Theory:** What methodological orientation was stated to underpin the study? e.g. grounded theory, discourse analysis, ethnography, phenomenology, content analysis	p. 7
**Participant selection**		
10	**Sampling:** How were participants selected? e.g. purposive, convenience, consecutive, snowball	p. 6
11	**Method of approach:** How were participants approached? e.g. face-to-face, telephone, mail, email	p. 6
12	**Sample size:** How many participants were in the study?	p. 8 & Supplementary table 1
13	**Non-participation:** How many people refused to participate or dropped out? Reasons?	N/A
**Setting**		
14	**Setting of data collection:** Where was the data collected? e.g. home, clinic, workplace	p. 6
15	**Presence of non-participants:** Was anyone else present besides the participants and researchers?	Supplementary table 1
16	**Description of sample:** What are the important characteristics of the sample? e.g. demographic data, date	p. 9 & Supplementary table 1

**Data Collection**		
17	**Interview guide:** Were questions, prompts, guides provided by the authors? Was it pilot tested?	p. 7
18	**Repeat interviews:** Were repeat interviews carried out? If yes, how many?	p. 7, 8 & Supplementary table 1
19	**Audio/visual recording:** Did the research use audio or visual recording to collect the data?	p. 7
20	**Field notes:** Were field notes made during and/or after the interview or focus group?	N/A
21	**Duration:** What was the duration of the interviews or focus group?	p. 8
22	**Data saturation:** Was data saturation discussed?	p. 19
23	**Transcripts returned:** Were transcripts returned to participants for comment and/or correction?	Supplementary table 1
**Domain 3: analysis and findings**		
**Data analysis**		
24	**Number of data coders:** How many data coders coded the data?	p. 7
25	**Description of the coding tree:** Did authors provide a description of the coding tree?	No
26	**Derivation of themes:** Were themes identified in advance or derived from the data?	p. 7 (derived from data)
27	**Software:** What software, if applicable, was used to manage the data?	N/A
28	**Participant checking:** Did participants provide feedback on the findings?	p. 7 & Supplementary table 1
**Reporting**		
29	**Quotations presented:** Were participant quotations presented to illustrate the themes / findings? Was each quotation identified? e.g. participant number	pp 8 - 15 & supplementary
30	**Data and findings consistent:** Was there consistency between the data presented and the findings?	pp. 16 - 19
31	**Clarity of major themes:** Were major themes clearly presented in the findings?	pp. 8 - 19
32	**Clarity of minor themes:** Is there a description of diverse cases or discussion of minor themes?	Supplementary file

* page number refers to page number of draft manuscript submitted for peer review.

Supplementary Figures

Figure S1: Indigenous groups representing distinct cultural, language, customs and laws of Central Australia,

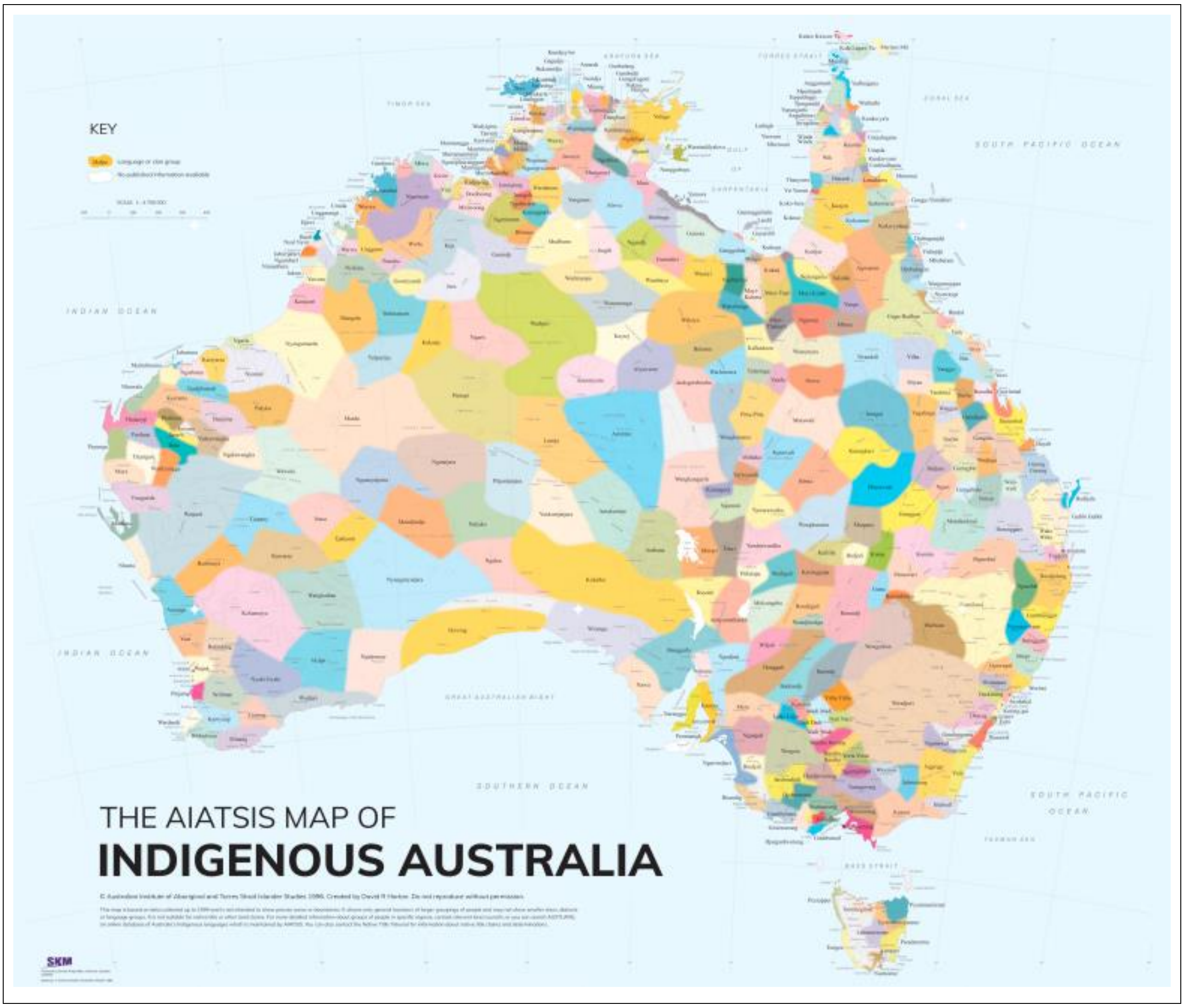

Disclaimer: *This map attempts to represent the language, social or nation groups of Aboriginal Australia. It shows only the general locations of larger groupings of people which may include clans, dialects or individual languages in a group. It used published resources from the eighteenth century-1994 and is not intended to be exact, nor the boundaries fixed. It is not suitable for native title or other land claims. David R Horton (creator), © AIATSIS, 1996. No reproduction without permission. To purchase a print version visit:* ***https://shop.aiatsis.gov.au/***

Reproduced with permission

Supplementary Detailed Thematic Analysis

---

1. A new topic and culturally taboo: It′s a really difficult topic to talk about

1.1. ORGAN DONATION IS A NEW TOPIC

Organ donation after death is a relatively new concept for Indigenous people in Central Australia to consider, including by the ALOs. Organ donation is also seen as incompatible with many Indigenous cultural practices and protocols around passing away. As noted by one of the senior ALOs,

> *In the past – when our people were walking around - we never had organ donation…it's never been spoken about in the past with our mob. For our people it's a new thing - [the] only organs we know is when we go out hunting that come from animals, it's a totally new thing.* (HW, FG 4, 2021)

During the early focus group discussions (FGD) there was some confusion between live donation and donation after death. The participants were familiar with the former and many personally knew of live kidney donors and the subsequent transplant recipient

> *In the past our family members were happy to help their cousin, sister or someone of the same blood type, to give them kidneys. That was happening in the 90s, donating a kidney to another family member.* (HW, FG 6, 2021)

The consensus was that organ donation is synonymous with receiving organs, particularly kidneys Where these organs came from and the concept of organ donation after death were seen as having been largely absent from the discourse. Unfamiliarity with the concept has resulted in families being confronted and overwhelmed when approached in ICU about donating a loved one's organ, especially at a time of grief.

> *[Talking about organ donation] it stresses us, it stresses our mob.* (HW, FG 2, 2021)

1.2. ORGAN DONATION IS TABOO

The concept of donating organs after death is confronting and taboo for most Indigenous people. The practice has been introduced by the "*white man's medical system and contrasts with Aboriginal ways of getting better and passing on*" (EL, 2021, pers. comm.) Significantly, the concept of donating organs after death was seen as confronting, juxtaposing with the Indigenous practice of return to country (the place they were born) and of being buried whole.

> *In rehla's (Aboriginal people's) way, he can't half ult-antama tinchultumay (a person can't be buried half empty). They take everything with them; they don't want to leave things here* (JA, FG 2, 2021).

> *Dying off country is like taking a book from a book shelf and never returning it … it leaves a hole that can never be filled.* (HW, 2021, pers. comm.)

The term "taboo" emerged on several occasions during the early focus groups, prompting further discussion around whether families should even be approached about the possibility of organ donation.

> *That's a taboo for me and my family and then that belief like our elder people always tell us you pass away you return back to this, by what dreaming, what totem you are you become that you know, that's who we are but when it comes to organ donation it's, you know, a very, very difficult discussion to have with our mob, very difficult because I can agree but when I'm dead they won't agree you know, things like that.* (HW,FG 1, 2021)

> *It's sort of taboo really amongst our people so we need to be careful about what we're treading on. (HW, FG 4, 2021)*

A senior ALO further explains by offering an anecdote about an amputee who requested to bury his leg in the place his body would later return to so that his spirit would be complete after death,

> *I remember coming across it in surgical ward with an old fella. Part of his leg was chopped off and he said, "No, no, no. No you can't take it. I've got to go back for a funeral". And I'm thinking, "What funeral"? He said, "I'm going to bury my leg first, have a funeral for my leg". Because when you look at it from the Aboriginal perspective our body is part of the land. Whatever we are going to be giving, [we are] giving away a piece of something which we relate to that land and when we die our body goes back to the land and our spirits roam free on the land. That's who we are.* (HW, FG 1, 2021)

One of the participants noted that her family's perspective on organ donation presented a challenge for her in accepting work in this area of medicine. In conversations with her elders,

> *They told me, "It's a tricky one, organ donation is not our thing", but on the other hand, some of them recognised the value in my involvement in finding ways to address the high rates of chronic disease and renal dialysis in our community.* (EL, FG 1, 2021)

This experience and that of other FGD contributors demonstrate that within the population there are varying attitudes to organ donation which can be influenced by age and traditional living practices.

1.3. CONFLICT BETWEEN "RELATIONALITY" AND ANONYMITY OF ORGAN DONATION

The accepted practice within the organ donation community of mandating anonymity and confidentiality for donors and recipients 'conflicts with the integrity of kin to Indigenous family and community life. The ALOs noted that when someone pursues a practice that "goes against" Indigenous culture such as organ donation, it can be used against them (sometimes referred to as 'payback') and be the cause of "*disagreements and*

fights" (HW, FG 1, 2021). This was of particular concern when considering the possibility of an organ being donated to a family that you may be in conflict with.

Sharing and supporting within family is the norm for Indigenous people who tend to live in very close proximity. Hence, altruism between family members is very common. This is sometimes referred to as demand-sharing.[2,3] However, altruism outside these family networks is less common. This reflects the reluctance of Indigenous people to donate their organs to strangers. Glen notes that, due to this, in his role as an ALO it would be *"very hard"* to tell a family they were *"giving away a kidney to a stranger"* (GA, FG 5, 2021)

The ALOs noted that if the recipient was related to the family of the donor, organ donation rates may increase. Glen points out that if organ donation occurred within family groups, then at least the organ would be returning to the same land following the death of the recipient,

> *… if that person can give it [organ] to an Aboriginal family member then that organ will still go back to the land, that's what I was thinking.* (GA, FG 3, 2021)

An important concern was lack of anonymity. In what are small Indigenous communities, someone receiving a transplant after the death of a community member may feel a responsibility to the immediate family of the donor.

1.4. CHRISTIAN CONCEPTS HAVE BEEN ABSORBED INTO TRADITIONAL KNOWLEDGE

There was an observation that Christian based religion was not part of traditional Indigenous culture, but that over time many of the concepts introduced by missionaries has been interwoven with Indigenous culture and has become accepted. Insofar as different denominations have different views of medical treatment (including donation and transplantation), there was an acknowledgement that this occurs.

(Y)ou know every Aboriginal community we preach God, unexpected death happens at home you know and it wasn't meant to happen. We see that family member pass away early in the morning when we wake up for breakfast and I know that I can say this and you know we all say it oh it was God's plan you know and we accept it, oh it was God's choice, she passed away in her sleep peacefully. (EL, FG 4, 2021)

"I know our mob we're coming back you know from generations from very, very strong Aboriginal communities and law and culture and all that but nowadays, today we're all about God you know, we pray and we pray." (EL, FG 4, 2021)

Yeah, we [medical staff] want to give you [a new] kidney and dialysis and she said, "No, Jesus told me not to look for dialysis". They [community members] were being told by the church not to go on dialysis because God told them not to. The Church tells them, "You can't go and get a kidney or anything like that because you're not supposed to look for medicine". Because of the church…she went and passed away and she was only in her 20s and she had a little baby and, you know, it's because of the church's influence (CS, FG 4, 2021)

2. Confronting conversations: Families might get angry if they get asked like that

2.1. BEDSIDE CONVERSATIONS: SHAMEFUL AND CONFRONTING FOR ABORIGINAL LIAISON OFFICERS AND FAMILIES

Due to the sensitive and taboo nature of the topic, many ALOs report avoiding direct involvement in initial conversations with families about organ donation in ICU. When they have been involved, it has mainly been with clinical staff who have conducted the initial conversations; however, from the ALO's'perspective this was not always done in a culturally safe way (see section 4.1). One of the ALOs describes her hesitancy in being on the front line of raising organ donation, especially with families from remote communities,

> *Families might get angry if they get asked for organs like that, especially when they are mourning. They would say, "Get out, what are you hanging around there for?"* (JA, FG 2, 2021)

> *They'd look at you thinking, "Are you mad, are you crazy?", [they might] come along with spears and spear us, boomerangs and spears* (LB, FG 3, 2021)

The current lack of understanding and knowledge about organ donation amongst families and communities within Central Australia has resulted in feelings of shame, confusion, offensiveness and confrontation when these families have been asked about organ donation in ICU. This has led to pressure and uncertainty for the families at a time of immense grief. It has also resulted in the direction of animosity, with resulting retribution, directed at the ALOs.

The ALOs expressed varying concerns about being part of these conversations, and more so if related to the family they were working with: "*really uncomfortable*" (CS, FG 3, 2021); a "*shame job*" (LB, FG 3, 2021); it would be so offensive to the families that "*(t)hey could bone us and make us very sick too, through witchcraft*" (LB, FG 3, 2021)). The ALOs were also concerned that their involvement in the conversations

could be seen as trying to promote organ donation and hence be aligning themselves with "*white man's*" medical practices.

2.2. SHOULD THE QUESTION BE ASKED?

Throughout the focus groups, the discussion regularly cycled back to a central question - "(S)hould Indigenous families, particularly those whose lives were largely orientated around traditional cultural practices, be asked about organ donation in ICU?" While it was acknowledged that everyone should have the choice, there was consensus that the current approach to asking Indigenous families was not culturally safe for two key reasons: First, the lack of existing education and understanding about organ donation amongst Indigenous families led to shame, confusion and offence amongst those families. Second, it was felt that the approach of some non-Indigenous clinical staff in communicating about organ donation was not undertaken in a culturally safe or acceptable manner. Hence, if the question was to continue to be raised at the bedside of loved ones, the ALOs encouraged a change in practice. There was a clear message that the current approach did not suit the context.

> *I think it's not on cause people get upset, especially in a sorrow (grieving) mode. They don't want to talk about people's body getting dismembered…so I think that ICU discussion with the doctors between the family and the patients, that needs to come to a halt at this stage. We need to educate them about organ donation prior [to arriving in ICU]. [Currently] you're catching people off guard when they're in sorrow mode and sometimes they're upset, and they don't want to make a decision* (HW, FG 6, 2021)

As well as the need for foundational understandings about organ donation prior to a terminal event in the ICU, the ALOs suggested the need to gauge a family's willingness to discuss organ donation before they were directly asked. If unwilling, the ALOs recommended that the conversation be taken no further; "*If they say no you just stop right there*" (Jenny, FG 6, 2021). The ALOs did feel that they had a role in being a conduit between clinicians and families during this distressing time, and that their in-depth knowledge of the

relational and cultural orientation of Indigenous families, provided them with unique insights into the timing and nature of these conversations.

2.3. DECISION MAKING AND SORROW TIME

The perceived urgency around organ retrieval was seen as obstructing the grieving process. For example, the apparent push by non-Indigenous clinical staff for families to arrive at a decision and to arrange the logistics around organ retrieval did not necessarily fit with the required time for families to travel to the hospital from remote communities, and to participate in cultural grieving practices or *"sorrow time"* which *"may be hours and hours"* (CS, FG 3, 2021). An anecdote highlighted this; One family had agreed to donate their loved ones 'organs, but when the clinical staff inferred "Hurry up we need to take the body" the family member became angry as she hadn't had time to complete her grieving and other family had not had adequate time to get to the hospital (CS, FG 1, 2021). The required expedited "sorrow time" for families in the event of organ donation also puts uncomfortable pressure on ALOs when they are involved in the transaction with families. This was exacerbated by a perceived pressure from medical staff (as noted by one ALO),

> *You see the donor lady there? [They are] hanging around like vultures waiting* (LB, Fg 3, 2021)

Gratuitous compliance (where people agree to consent even when they do not agree or do not fully understand what they are agreeing to) is a serious risk in a system such as the ASH where there are cultural misunderstandings, language barriers and significant power differentials between the medical staff, patients and their families.[4] One ALO described a situation where a family member had initially agreed to organ donation. However, she had not been informed of the shortened timeframe she would have to grieve before the body was taken away

> *She said yes. She got a little bit of education on it but then when it was time, she said, "No, no, no don't take her yet we're not ready yet". She was waiting for an aunty to come from Tennant Creek. The organ donor mob was there and they were like "Hurry up we need to take the body". That*

made her angrier and she said, "No, no you're not taking my sister" and she just stopped [and withdrew her consent]. (CS, FG 1, 2021)

As well as organ donation hastening the grieving period, the ALOs drew attention to the need for the system to provide adequate time and space to enable families to follow decision-making protocols which are culturally safe and inclusive of the required family members. Inevitably, the dearth of knowledge around organ donation and the many "next of kin" extends the required time for decision-making.

> *[Medical staff ask], "Who is your next of kin?" Well, I've got 50 cousins on this side, another 20 on this side, who's going to be fighting over to make a decision around my organs? You know, so [it's difficult] to have both sides on the same page as well. (HW, FG 2, 2021)*

There was a common misunderstanding amongst non-Indigenous colleagues that Indigenous patients have only one "next of kin",

> *For Aboriginal families there is not just one next of kin. I tell those doctors at ICU, "Don't speak to one family member, you're going to need to talk to the father's side and then the mother's side and to grandparents and the cousins.* (EL, FG 2, 2021)

Extended next of kin of patients is further complicated by the cultural and social hierarchy existing in family groups, an intricacy that many non-Indigenous staff are not familiar with, and if correct protocol is not followed during decision-making around death there can be serious ramifications for family members.

Too often, ALOs noted, there is an expectation by non-Indigenous clinical staff that choices be made as quickly as possible and incorrect family members are invited to make the decisions. This problem could be resolved by closer consultation with the ALOs given their knowledge of Indigenous family dynamics and relationships. For example, both the "*mother's and father's side*" of the family need to be represented in the discussion (CS, FG 3, 2021). EL explains a situation in her family which indicates the importance of

everyone having an opportunity to have a say. A patient and her family members had agreed to organ donation however there was one elder who objected and hence, it couldn't go ahead,

> *It was a "yes" from the patient and the other side of the family but then it came back to one elder in the family, that elder said "No". So we couldn't get organs out of this young lady because of that one person. She had the power to say no and everybody agreed.* (EL, FG 2, 2021)

Based on current practices in the ASH ICU, the ALOs did not feel comfortable about being involved in family conversations about organ donation due to retribution, shaming and the likelihood that they would be causing additional grievance for families during "sorrow time". They noted three essential requirements going forward:

1. Appropriate resources and community education to inform the conversations;
2. Better understanding by non-Indigenous staff about decision-making protocols for Indigenous families;
3. Indigenous-informed processes for decision-making such as purposeful family meetings with appropriately identified family members.

3. Education about organ donation: *Our people need to know*

3.1. DESIGNING APPROPRIATE RESOURCES

Reflecting on their own evolving knowledge about organ donation, the ALOs noted an urgency to develop a set of culturally and linguistically appropriate resources to inform Indigenous audiences about organ donation. If families were equipped with a basic knowledge of organ donation after death prior to being approached in ICU, the discomfort for families would be minimised and the ALOs would *"feel good and comfortable"* about supporting the decision-making process (LB, FG 3, 2021). Currently, the lack of foundational knowledge means the initial conversations about organ donation are complex, confrontational and imbued with cultural misunderstandings. The need for prior education was emphasised,

> *The conversation starts within the community itself way before coming to ICU. You've got to start educating…At the moment there are no resources out there to [support us to] talk about it.* (HW, FG 4, 2021)

The ALOs suggested a range of place-based and locally developed resources: brochures, flip charts, information sessions, posters and videos; ideas inspired in part by reviewing resources developed for Indigenous audiences in other parts of Australia. The ALOs recommended a range of audiences and locations for sharing these resources: community clinic waiting rooms, interagency-supported community events, the Alice Springs Hospital television channel, apps for smart phones, social media and local television stations (Indigenous Community Television (ICTV) and National Indigenous Television (NITV)). They noted the importance of the role of community-based health staff in disseminating this information so that people can learn *"on country"* (LB, FG 3, 2021). All resources would need to be translated to be accessible by a linguistically diverse population.[1]

Education in organ donation needed to be interwoven with messages about overall wellbeing so as to ultimately minimise the need for organ transplants. Of particular concern to the ALOs was easy access to

[1] As a consequence of this research, the project team successfully applied for a DonateLife Community Awareness Grant and a series of animated videos about organ donation in multiple languages is currently in production under the guidance of the ALOs.

unhealthy fast food in Alice Springs which was described as, *"ordering death"*. It was noted that the popular take-away stores run by non-Indigenous businesses lead to *"blocked arteries, kidney problems, diabetes, fatty livers, obesity and kids as young as ten on dialysis"*, and that it is a way of government*" killing our mob, something they've been trying for 223 years"* (HW, FG 4, 2021).

3.2. BUSTING MYTHS ABOUT ORGAN DONATION

The ALOs highlighted the need to address myths about organ donation. Some could partly be ascribed to popular media, but that lack of appropriate and accessible information for Indigenous people was the main problem. These myths perpetuate scepticism and misunderstanding about organ donation. For example;

> *You read [in a magazine] that when you take on somebody else's organ you take on the personal traits of the person that you received it from. One smoker might become a smoker or start drinking beer when they weren't beer drinkers, things like that.* (CS, FG 1, 2021)

Additionally, myths were linked to complicated medical terminology. For example, it was pointed out that there may be issues with common medical terminology;

> *…and that word "transplant", they're thinking they're getting another plant in them, we need to change it to straight out and say, "You're going for a new kidney"* (HW, FG 6, 2021)

As well as busting myths, the ALOs pointed out some significant facts they learnt about organ donation within the FGDs which were critical to consider in the development of resources for the community:

1. Organ donation can significantly increase life expectancy;
2. Multiple recipients can benefit from the organs of one deceased person (one organ and tissue donor can transform the lives of 10 or more people);[5]

3. Because of the high rates of chronic disease, many Indigenous people are led to believe that they have sick organs simply by virtue of being Indigenous. However, people need to understand that even heavy drinkers or smokers may be eligible organ donors as many different organs can be donated;
4. A better understanding about blood compatibility and the medical process of organ donation, and;
5. The link between donation and transplantation. Currently there is a much greater awareness and familiarity with receiving organs but little understanding about their origin.

3.3. CHANGES IN ATTITUDE OVER TIME

The ALOs explained that with appropriate and sensitive sharing of education and resources, Indigenous attitudes to organ donation may change over time from, *"No, I don't want someone else's body parts in me"* to an acceptance of organ donation (HW, FG 2, 2021). One of the ALOs likened the increased understanding about organ donation to other big shifts in community thinking about medical interventions such as the gradual acceptance of blood transfusions *"back in the 80s"*. There was once concern that,

> *…having somebody else's blood could make them sick. It scared the whole community…[but] they accept it now [that there is better information about it].* (CS, FG 6, 2021)

It was noted that adequate resources and education might lead to an increased trust in the medical system and hence, *"the next generation"* may be more willing to *"talk about organ donation"* and turn to procedures that prolong life (CS, FG 6, 2021; a choice that Indigenous people have the right to understand so they can *"have a second chance to enjoy life"* (HW, FG 4, 2021). Additionally, the ALOs pointed out that with increased education individuals might be more likely to*" sign up"* for organ donation on the national registry which would assist some families with making the decision at the bedside in ICU.

4. Cultural education (of the western medical system): They work in a square, we work in a circle

4.1. CULTURAL SAFETY AND CULTURAL AWARENESS OF ICU MEDICAL STAFF

A lack of sensitivity and cultural awareness by non-Indigenous staff in ICU was a factor that contributed to the topic of organ donation being confronting and uncomfortable for Indigenous families, patients and staff. Despite nearly 70% of the clinical case load of the ASH ICU being Indigenous, there are no Indigenous intensivists, and very few Indigenous nursing staff. Many of the junior medical staff are rotating staff undertaking ICU terms as either part of other specialist training programs (Emergency Medicine, Anaesthetics) or are intensive care trainees rotating from interstate hospitals. Although efforts are made to orientate all staff to some of the intricacies of managing patients from very different cultural and social backgrounds, there is incomplete penetration, and limited exposure. Rather, their understanding of Indigenous families primarily stems from their limited experiences in the health care system where non-Indigenous cultural frameworks prevail. The ALOs were particularly concerned about the way non-Indigenous medical staff talked about death.

It is difficult to talk about deceased organ donation without talking about death. However, death is a very sensitive topic for Indigenous people. Using the English words death, dying or died (that came to be referred to in the focus groups as "The three Ds") is considered confronting and offensive. As stated by one ALO, *"when medical staff use that language when the families are going through a really hard time it makes it harder for them"* (CS, FG 5, 2021). To minimise the pain for families and to enhance cultural sensitivity, the ALOs say that end of life should be referred to in *"roundabout ways"* (EL, FG 6, 2021).

> *We would say something like, "Sorry but the doctors can't do anything for you."* (CS, FG 5, 2021)
>
> or
>
> *"Sorry but the medicines can't help anymore". We (ALOs) interpret that back to the patients or families [in ways that are comfortable for them].* (CS, FG 6, 2021)

The term *"organ donation"* was also thought to be problematic as it sounds very *"harsh and cruel"* when translated into Indigenous languages (CS, FG 1, 2021). This places the ALOs in a compromising position when they are interpreting for medical staff and expected to *"say exactly what comes out of the doctor's mouth"* (HW, FG 5, 2021). However, when given the autonomy to manage this discussion with families, the ALOs can adopt less direct, more gentle *"watered down"* (HW, FG 5, 2021) ways of talking about organ donation and end of life that builds rapport, elicits questions from family members and minimises additional stress for families when they are *"in sorrow"* (HW, FG 5, 2021).

The ALOs pointed out that the barriers to communication in ICU were not just attributable to cultural unfamiliarity, and wondered whether there was also an element of racism. They thought it was unlikely medical staff would talk to many non-Indigenous patients in the way they talk to Indigenous families about sensitive matters like death,

> *They don't talk like that to the white fella families, white family they'll use some other words, not straight out, "You're going to die"* (LB, FG 5, 2021)

4.2. NEED FOR INCREASED RECOGNITION OF ABORIGINAL LIAISON OFFICERS AS CULTURAL EXPERTS

Poor or offensive communication between clinical staff and Indigenous patients/families leads to scepticism about medical services and advice. For example, following a bad experience a patient shared with one ALO their concern that *"they [the medical staff] are going to poison me here"* (CS, FG 6, 2021). Patients 'and families 'scepticism of non-Indigenous medical staff puts immense pressure on the ALOs to provide them with reassurance, support, interpretation and cultural safety in an environment where many feel alienated and disempowered.

The ALOs describe that their rapport with patients is largely influenced by the way they position themselves as workers which doesn't always correspond with non-Indigenous cultural parameters of "work". For example, there is a blurred line between work and family life for the ALOs. In other words, the centrality of relatedness for Indigenous people means that their work as ALOs doesn't just occur within their paid hours

of work. An ALO described this as a metaphor of squares (non-Indigenous workers) and circles (Indigenous workers), whereby their work as ALOs is circular; it never stops and is strongly interwoven with their family and community commitments,

> *The mentality of Aboriginal people is a circle. A white man's perfect world is a square, our circle doesn't fit into it, you know, it's just square - they come to work 6AM, knock off 4:21PM. They use square phones, square laptops, everything is square and when they knock off they go back to the square inside the yard, inside the house. We, we're still in a circle, you know, when I knock off we go and sit down with family, we mix up with family, we still meet the patients out on the street, we deal with it that way every day everyday.* (HW, FG 6, 2021)

Another ALO further described the overlap between work and home life,

> *After work we've still got sorry business. After being with people in ICU, they still come to us with questions like, "Where can I go?" or "What can I do for my family member?". They come [to my] home knocking on my door. Our work keeps going after work* (CS, FG 6, 2021)

The ALOs drew attention to how challenging it can be for them to navigate the two worlds in which they work, including the struggle for them to pass on western medical information and advice in a way that aligns with the cultural views of Indigenous patients. Often there is a clash between these world views. The medical "evidence" passed on from the medical staff to the ALOs often means little to the patients and their families. For example, the ALOs understand that the *"numbers on the computer"* (EL, FG 6, 2021) equate to the patient having diabetes, but the patient's response is, *"I don't feel sick, I'm feeling all right"* (CS, FG 3, 2021) and it can be *"really difficult for them to accept"* (EL, FG 6, 2021). In the ALOs 'experience, the patients may have a solution other than non-Indigenous medical intervention, such as consulting a Ngangkari, a traditional healer of the remote western desert,[6]

Doctors can look at it [results] and say, “Blood tests were done so we took a scan and we found this tumour in you and I think it’s cancer”, but sometimes they (the patient) looks at it and says, “No, a witchdoctor will fix that”, you know, they think like that that. (HW, FG 6, 2021)

Despite the centrality of their role in supporting and interpreting for patients and families navigating a largely foreign system, ALOs noted that many medical staff do not understand their role and the expertise they can provide during medical transactions. Hence, clinical teams often fail to utilise them in ways that could enhance rapport with patients. This approach ultimately impacts on treatment and health outcomes. An ALO provides an example whereby his advice about holding back from asking a family about organ donation was ignored and this resulted in jeopardising his relationship with the family,

[The conversation] took place and the partner got upset about it because he didn’t understand organ donation…and he said, “No, no I’m not doing this”. I advised the doctor not to ask…I said, “Don’t go down asking these questions, just leave it at this cause they’re a bit sad”. But it still happened and the patient’s partner got upset…he’s still upset today about it when I seen him. It puts us Liaison Officers in between, where we’re not getting trusted. You know, our job is about trust building. (HW, FG 6, 2021)

The ALOs made it clear that if they were recognised and treated as cultural experts – not just interpreters – by clinical staff then confrontation and mistrust were less likely and the experiences of everyone in in the health system could be improved. They also suggested that clinical staff could improve their understanding of their patients ’world by occasionally stepping out of their square and into the circular world of Indigenous people i.e. by attending sorry business and *“give their respect”* (CS, FG 6, 2021).

5. Mistrust: *That's why community doesn't trust them*

On a number of occasions, the theme of mistrust of institutions emerged, and in particular the mistrust around the motives of government, churches and the health care system. This appeared to spill over into concerns about the motives of government programs and policies, with the underlying assumption that there was always a hidden agenda that would further disadvantage Indigenous people.

> *That's where all government thinking is wrong. I think they've been trying to kill us off for 223 years you know. They've found a way of killing our mob. They're saying, "All right, let's put all these takeaway shops here, and put more sugar drinks in the shops so these kids can grow up with diabetes, you know, then kill them off like that and [ensure] they've got short expectancy of life…That's what's happening, they're killing us off.* (HW, FG 4, 2021)

> *Churches have been helping the government to steal the land so what's the point you know. That's how I see it. They came along and our people put their head down and prayed and turned around and looked and they, hey where's my land, it's gone, you know? And they were told to work 14 hour shifts to build a yard, build a fence line and next minute they kicked over the other side of the fence line and said you live on this side.* (HW, FG 4, 2021)

> *(T)hat's why they don't have that trust with the community or community doesn't trust them because of the issues in the past or you know.* (CS, FG 6, 2021)

> *The government says oh you can't spend your money, I'm going to quarantine half your money into a Basics Card the Basics Card is supposed*

to be for about health. Now you've going to run around right there at Hungry Jacks and KFC you can use your Basics Card you know. (HW, FG 4, 2021)

It was thought that this mistrust about organ donation could possible be mitigated through the use of education, especially if it was culturally and linguistically appropriate and had local input into its design.

Not just when you're sick you need to, you know educate people before, it could happen with a young person who is, it might happen you know through the night coming into town. Educate from the community, we've got to educate them. It will happen you know, you never know. Just like that it will happen or something. (BS, FG 1, 2021)

References

1. Tong A, Sainsbury P, Craig J. Consolidated criteria for reporting qualitative research (COREQ): a 32-item checklist for interviews and focus groups. *Int J Qual Health Care*. Dec 2007;19(6):349-57. doi:10.1093/intqhc/mzm042
2. Peterson N. Demand Sharing: Reciprocity and the Pressure for Generosity among Foragers. *American Anthropologist*. 1993;95(4):860-874. doi:https://doi.org/10.1525/aa.1993.95.4.02a00050
3. Beadle R. Remote Aboriginal women and meaningful work: key dimensions for Ngaanyatjarra women. 2018. Accessed 8 Feb 2022. https://minerva-access.unimelb.edu.au/items/e77a2cbf-c5c7-5b18-be8a-6c26753ffce1
4. Liberman K. Ambiguity and gratuitous concurrence in inter-cultural communication. *Human Studies*. 1980/12/01 1980;3(1):65-85. doi:10.1007/BF02331801
5. Transplant Australia. The Facts. Transplant Australia; 2019. Accessed 14 Mar 2023. https://transplant.org.au/the-facts/
6. NPY Women′s Council. Ngangkari – Traditional Healers. Accessed 08 Feb 2023. https://www.npywc.org.au/what-we-do/ngangkari-traditional-healers
